# ADIPOQ gene (T45G and G276T) single nucleotide polymorphisms and their association with gestational diabetes mellitus in a Filipino population

**DOI:** 10.1186/s12902-023-01479-z

**Published:** 2023-11-13

**Authors:** Larah Francesca A. Fernandez, Maria Ruth B. Pineda-Cortel

**Affiliations:** 1 The Graduate School, University of Santo Tomas, Manila, Philippines; 2 Research Center for the Natural and Applied Sciences, University of Santo Tomas, Manila, Philippines; 3 Department of Medical Technology, Faculty of Pharmacy, University of Santo Tomas, Manila, Philippines

**Keywords:** Gestational, Diabetes, Adipocytes, Adiponectin, Polymorphism, Risk, Pregnancy

## Abstract

Several studies have associated the presence of ADIPOQ gene polymorphisms with insulin resistance, adiponectin levels, and metabolic diseases such as diabetes, although with varying degrees of correlation depending on ethnicity. Here we aim to identify individual’s susceptibility to gestational diabetes mellitus (GDM) in the presence of T45G and G276T single nucleotide polymorphisms (SNPs) within the ADIPOQ gene among Filipino pregnant women. A total of 285 pregnant women (95 GDM cases and 190 controls) were included in this study. Two ADIPOQ gene polymorphisms were genotyped using TaqMan assay. Results of SNP genotyping showed no significant differences in the frequencies of TT, TG and GG genotypes of T45G SNP between the GDM and control group [p = 1.0000, 0.6179, 0.5797; OR (95%CI) = 1.030 (0.582–1.874), 1.135 (0.683–1.828), 0.833 (0.481–1.420)]. Similarly, the frequencies of GG, GT, and TT genotypes of G276T SNP were comparable in both groups [p = 0.8002, 1.0000, 0.3466; OR (95%CI) = 1.090 (0.654–1.785), 1.022 (0.616–1.665), 0.433 (0.092–1.698)]. Moreover, although adiponectin levels were significantly decreased in GDM group (p = 0.0196) and have shown substantial negative correlations with FBS, 1-hour OGTT, 2-hour OGTT, and HOMA-IR (p < 0.05), they were not significantly different according to genotypes of T45G and G276T polymorphisms both in GDM and control group. Our results suggest that neither of the two ADIPOQ gene polymorphisms influence adiponectin levels and development of GDM in a Filipino population.

## Introduction

Gestational diabetes mellitus (GDM) is a condition of hyperglycemia observed in some pregnant women that is characterized by insulin resistance resulting to glucose intolerance [1, 2]. Although the condition is transient, it is linked to a variety of adverse maternal and fetal outcomes and the risk of developing Type 2 diabetes mellitus (T2DM) later in life, making it a public burden [3, 4]. This condition has significantly contributed to the vicious, long-standing sequence of obesity and diabetes that impacts the health of the general population [5]. It was estimated that about 20.9 million women have hyperglycemia during pregnancy equivalent to a prevalence rate of 16.2% and about 85.1% of these cases are due to GDM [6]. The rate of development increases with maternal age, varies in races/ethnicities, and is often intensified by obesity. Aside from these commonly identified risk factors of GDM development, evidences have been found that susceptibility to GDM has a genetic component. Various genetic variations or polymorphisms in several genes have been found to be associated with the risk of developing GDM. rs266729 of ADIPOQ gene, rs10811661 of CDKN2A/2B gene, rs9505118 of SSR1-RREB1, rs7177055 of HMG20A, rs16927668 of PTPRD, rs1535500 of KCNK17 gene to name a few [4, 7].

Variations in the results of genetic association studies have been identified depending on population being studied. Although individual studies on Malaysian [8], Chinese [9], Iranian [10], and Han [11] populations have consistently correlated G allele and TG/GG genotype of T45G SNP of ADIPOQ gene with higher risk of GDM, findings in the recent study by Nezamzadeh, et al. on Iranians refuted these results, concluding that T allele was the risk factor for GDM [12]. Contrary, other studies conducted on Arabs [13] and Bulgarians [14] were unable to find any correlation between T45G SNP and GDM. On the other hand, separate reports with regards to the association of G276T SNP of ADIPOQ gene with GDM conducted on Arabs [13], Bulgarians [14], and Caucasians [15] have consistently shown lack of correlation. However, several individual studies have also correlated this SNP with insulin resistance [16, 17], which is a hallmark of GDM, and type 2 diabetes mellitus (T2DM) [18, 19], yet conflicting results have been obtained even on studies conducted on similar ethnic groups.

Reports with regards to the correlation of other genetic polymorphisms with GDM also vary among various population groups. Particularly, association studies about rs266729 of ADIPOQ gene have shown variable results as to whether G allele is a risk factor for GDM [12, 15] or it can actually confer protection against the condition [14]. Previous studies about rs10811661 of CDKN2A/2B gene and GDM also indicated irregularities as other studies have established correlation [20, 21], while one study failed to identify any significant findings [22]. In another study regarding the association of rs7178572 of HMG20A gene with GDM, significant correlation was found among Indian population while no evident association was shown among the Scandinavian group [7]. Variable results on studies about rs2237892 of KCNQ1 gene and its contribution to GDM risk were also observed among various and even within the same population groups [23–26].

In this present study, our focus is the genetic variation in the ADIPOQ gene, particularly T45G in exon 2 and G276T in intron 2, which have also shown significant correlation with insulin resistance, adiponectin levels, and metabolic diseases although results still vary among different ethnic groups [8, 27–32]. ADIPOQ gene, which is located on chromosome 3q27, the region that was proposed to be a “susceptibility locus for type 2 diabetes” by the recent human genome-wide scans [32], encodes for the adipose tissue-derived adiponectin hormone [33]. This hormone plays a role in glucose utilization and fatty acid metabolism by activating the 5’-AMP kinase enzyme [34]. Studies have shown that adiponectin levels are negatively correlated with obesity, insulin resistance, metabolic syndrome, as well as T2DM [33]. Moreover, several studies also have demonstrated that adiponectin levels in early pregnancy are predictive of GDM progression in the later stage of gestation [8, 35, 36]. But as with other genetic association studies, results vary based on population being investigated [16, 37]. With increasing prevalence of GDM in the Philippines [38], identification of possible genetic component on the susceptibility to GDM is relevant. Here we aim to identify the association of T45G and G276T SNPs within the ADIPOQ gene with GDM among Filipino pregnant women, including genotype distributions and associations with adiponectin levels, glycemic parameters as well as insulin resistance.

## Materials and methods

### Subjects

All 285 pregnant women included in this study were of Filipino descent and recruited from a tertiary hospital in Metro Manila, Philippines, from 2018 to 2020. Our study conforms to the Helsinki declaration. The Institutional Review Board of University of Santo Tomas Graduate School, Manila approved the study protocol as part of the project entitled, “Blood and placental gene expression in gestational diabetes mellitus: potential identification of early biomarkers” (E-2016-02-R3). All subjects in the study provided informed consent. Subjects were recruited following the study’s inclusion and exclusion criteria: pregnant women conceived spontaneously (not conceived with the help of Assisted Reproductive Technology – ART) within 9th week to 12th week of gestation, between 25 and 45 years of age (GDM risk progressively increased from age 25 years onwards) [39], free of recognizable diabetes, **without** history of GDM during their previous pregnancies, and without any of the following conditions: polycystic ovarian cyst, hypertension, kidney diseases, hepatitis, HIV, any bacterial or viral infection, and inflammation. Diagnosis of GDM was done using 75 g oral glucose tolerance test (OGTT) as requested by their attending physicians either during their second or third trimester of pregnancy. Diagnosis followed the International Association of the Diabetes and Pregnancy Study Group (IADPSG) as recommended by the World Health Organization (WHO) IADPSG criteria, that is, meeting one or more of the following thresholds: fasting blood sugar (FBS) of 5.1–6.9 mmol/L; first hour blood glucose of ≥ 10 mmol/L; and, second hour blood glucose of 8.5–11.0 mmol/L.

### Clinical measurements

Aside from OGTT, other clinical parameters were assessed. Total cholesterol, triglyceride, and HDL-c (high density lipoprotein-cholesterol) were measured using enzymatic methods. While VLDL-c (very low density lipoprotein cholesterol) was computed using the formula, triglyceride in millimoles per liter divided by 2.2, and LDL-c using the Friedewald formula, total cholesterol minus HDL-c minus VLDL-c. Briefly, total cholesterol was determined after enzymatic hydrolysis and oxidation using cholesterol esterase, cholesterol oxidase, and peroxidase. Using an indicator dye, 4-aminoantipyrene, quinoneimine was produced and measured spectrophotometrically at 500 nm. Similarly, triglyceride was measured following hydrolysis using lipase to release glycerol and fatty acids. Glycerol in the presence of glycerol kinase formed glycerol phosphate. This is then oxidized using glycerol phosphate oxidase to release hydrogen peroxide, which is reacted with 4-aminoantipyrene to release a colored product, quinoneimine. The product was measured spectrophotometrically at 500 nm. Glycosylated hemoglobin was measured using Nycocard™ reader (Abbott) following the principle of boronate affinity assay, wherein the glycated hemoglobin was bounded to a blue boronic acid conjugate following lysis of erythrocytes caused by the lysis reagent. The ratio of the bound (glycated) and unbound (total) hemoglobin was then measured and results obtained were proportional to the percentage of glycosylated hemoglobin in the sample. Adiponectin and insulin level determination was done using enzyme linked immunosorbent assay kits following the manufacturer’s protocol (BioLegend’s ELISA MAX™ Deluxe Sets and RayBio Human Insulin ELISA Kit, respectively). Insulin resistance was evaluated using the homeostasis model of insulin resistance (HOMA-IR) calculated using the formula, insulin in micro-International Units per milliliter multiplied by glucose in millimole per liter divided by 22.5.

### SNP genotyping

Two polymorphisms of ADIPOQ gene previously reported in other studies were selected and genotyped using TaqMan assay. Genotyping of T45G and G276T SNP of ADIPOQ gene was performed using the following context sequences: T45G:TTCTACTGCTATTAGCTCTGCCCGG[G/T]CATGACCAGGAAACCACGACTCAAG (C_26426077_10) and G276T:CTACACTGATATAAACTATATGAAG[G/T]CATTCATTATTAACTAAGGCCTAGA (C_7497299_10). Quality control was performed in 20% (n = 57) of the samples by duplicate checking, and rate of concordance should be > 99%.

PCR amplification and genotyping were performed in a total reaction volume of 10uL reaction mixture containing: 5uL of TaqMan Universal Mastermix II (2x), 0.5uL of TaqMan SNP Genotyping Assay (20x), and 4.5uL of DNA eluate. Thermal cycling conditions used were: polymerase activation (92 °C for 10 min) and 45 cycles of denaturation (92 °C for 30 s), and annealing (60 °C for 90 s). Results were analyzed on Rotor Gene Q System Software.

### Data processing and statistical analyses

Respondents were classified into control and GDM groups according to their OGTT results and physician’s diagnosis. Continuous characteristics including age, height, weight, body mass index (BMI), fasting blood sugar (FBS), lipid profile, insulin, HbA1c, HOMA-IR, and adiponectin levels were presented as mean +/- standard error of mean (SEM). Mann-Whitney *U* test analysis was used to compare the means of the summarized clinical characteristics of the two groups (control and GDM groups). Relationship of the different genotypes and alleles of T45G and G276T SNPs with adiponectin levels and other biochemical parameters were determined using Point Biserial Correlation analysis. Analysis of the relationship of T45G and G276T SNPs with GDM was conducted using Fisher’s Exact test. Logistic regression analysis was used to calculate the odds ratio (OR) and 95% confidence intervals (CI) to determine the strength of association between ADIPOQ gene SNPs and GDM and to find out the magnitude in which a given exposure affects the specific outcome of interest. Additionally, genotyping results of both ADIPOQ gene polymorphisms were also assessed for Hardy-Weinberg equilibrium (HWE) in order to detect any possible genotyping errors. All statistical tests were performed using SPSS 20.0 and Prism GraphPad Version 8.2.1.

## Results

### Clinical characteristics of study sample participants

The clinical characteristics of NGT (normal glucose tolerance) and GDM participants are summarized in Table 1. The age and weight of participants with GDM were significantly higher than those participants with NGT. Glycemic parameters including FBS, 1 h OGTT, 2 h OGTT, and HbA1c were markedly elevated in GDM group. Similarly, increased HOMA-IR index was also observed in GDM group although no significant differences were found in terms of their insulin levels. On the other hand, adiponectin levels were significantly lower in GDM group compared to NGT group. The two groups however, did not differ significantly in terms of height, BMI, and lipid parameters including total cholesterol, triglyceride, HDL-c, LDL-c, and VLDL-c.

**Table 1:** Clinical characteristics of participants

Characteristic	NGT Group (Mean ± SEM) n = 190	GDM Group (Mean ± SEM) n = 95	*P*-value
Age (years)	25.37 ± 0.47	27.67 ± 0.69	**0.0058**
Weight (kg)	59.53 ± 1.01	63.53 ± 1.36	**0.0194**
Height (m)	1.55 ± 0.01	1.56 ± 0.01	0.6151
BMI	24.78 ± 0.44	26.29 ± 0.65	0.0622
FBS (mmol/L)	4.22 ± 0.04	5.53 ± 0.11	**< 0.0001**
OGTT 1st hour (mmol/L)	6.64 ± 0.10	8.49 ± 0.24	**< 0.0001**
OGTT 2nd hour (mmol/L)	5.69 ± 0.08	7.69 ± 0.23	**< 0.0001**
Total Cholesterol (mmol/L)	5.58 ± 0.13	5.87 ± 0.23	0.3453
Triglyceride (mmol/L)	3.46 ± 0.11	3.52 ± 0.21	0.8689
HDL-c (mmol/L)	1.85 ± 0.03	2.01 ± 0.12	0.4035
LDL-c (mmol/L)	2.22 ± 0.11	2.39 ± 0.21	0.6691
VLDL-c (mmol/L)	1.57 ± 0.05	1.60 ± 0.09	0.8668
HbA1c (%)	5.10 ± 0.04	5.39 ± 0.10	**0.0270**
Insulin (uIU/mL)	11.58 ± 1.73	32.40 ± 10.20	0.2237
HOMA-IR	2.17 ± 0.32	7.82 ± 2.41	**0.0004**
Adiponectin (ng/mL)	10.00 ± 0.08	8.92 ± 0.26	**0.0198**

*Significant if *P-*value is less than 0.05 (*p* < 0.05) using Mann-Whitney *U* test.

Legend: NGT, normal glucose tolerance, GDM, gestational diabetes mellitus, SEM, standard error of mean.

### Association of ADIPOQ gene T45G SNP with GDM

The genotype distribution of ADIPOQ gene T45G SNP did not deviate from their estimated frequencies and thus, is in agreement with HWE *(P* = 0.635). Genotype and allele frequencies in the GDM and NGT groups are represented in Table 2. Based on the results, the frequencies of TT, TG and GG genotypes as well as T and G alleles of T45G SNP showed no significant differences between the GDM and NGT groups (p > 0.05). Odds ratio was also computed and although negative association can be observed between GG genotype and GDM, this correlation was not statistically significant (OR (95%CI) = 0.833(0.481–1.420), *P* = 0.580). Results remained insignificant even after confounding factors such as age, weight, and BMI were removed (data not shown).

**Table 2:** ADIPOQ T45G Genotype and Allele Frequency Distribution in Gestational Diabetes Mellitus Patients and in patients with Normal Glucose Tolerance

Genotype	GDM		NGT			*P*-value	OR* (95%CI)
	n	%	n	%	HWE*		
**T/T**	22	23.16	43	22.63	0.635	1.000	1.030 (0.582–1.874)
**T/G**	48	50.53	90	47.37		0.618	1.135 (0.683–1.828)
**G/G**	25	26.32	57	30.00		0.580	0.833(0.481–1.420)
**T Allele**	70	73.68	133	70.00		0.580	1.200(0.704–2.079)
**G Allele**	73	76.84	147	77.37		1.000	0.971 (0.534–1.719)

*HWE, Hardy-Weinberg Equilibrium; *OR, Odds Ratio, NGT, normal glucose tolerance, GDM, gestational diabetes mellitus; Significant if *P-*value is less than 0.05 (*p* < 0.05) using Fisher’s Exact Test.

### Association of ADIPOQ gene G276T SNP with GDM

The genotype distribution of ADIPOQ gene G276T SNP was also in accordance with HWE in the NGT group (*P* = 0.102). Genotype and allele frequencies in the GDM and NGT groups are shown in Table 3. Similar with the results of T45G SNP, the frequencies of GG, GT, and TT genotypes as well as G and T alleles of G276T SNP were comparable in both groups (*P* > 0.05). Negative association can also be noticed between TT genotype and GDM, however this observation does not reached significant statistical data (OR (95% CI) = 0.433 (0.092–1.698), *P* = 0.347). Same findings were indicated even after confounding factors were removed (data not shown).

**Table 3:** ADIPOQ G276T Genotype and Allele Frequency Distribution in Gestational Diabetes Mellitus Patients and in patients with Normal Glucose Tolerance

Genotype	GDM		NGT			*P*-value	OR* (95%CI)
	n	%	n	%	HWE*		
**G/G**	55	57.89	106	55.79	0.102	0.800	1.090 (0.654–1.785)
**G/T**	38	40.00	75	39.47		1.000	1.022 (0.616–1.665)
**T/T**	2	2.11	9	4.74		0.347	0.433 (0.092–1.698)
**G Allele**	93	97.89	181	95.26		0.347	2.312 (0.589–10.83)
**T Allele**	40	42.11	84	44.21		0.800	0.918 (0.560–1.530)

NGT, normal glucose tolerance, GDM, gestational diabetes mellitus; *Significant if *P-*value is less than 0.05 (*p* < 0.05) using Fisher’s Exact Test.

### Association of ADIPOQ gene T45G and G276T SNPs with adiponectin levels

We also examined the relationship between ADIPOQ gene polymorphisms (T45G and G276T) and adiponectin levels to find out whether they directly affect the hormone’s blood levels. Although adiponectin levels were significantly reduced in GDM group (8.92 ± 0.26 vs. 10.00 ± 0.08; *P* = 0.0198), no significant differences in the levels were found between TT and TG/GG genotypes of T45G and GG and GT/TT genotypes of G276T SNPs (*P* = 0.193, 0.661) (Table 4).

**Table 4:** Comparison of mean adiponectin level between NGT and GDM group and among participants with different genotypes of T45G and G276T SNPs in ADIPOQ gene

Group	Adiponectin (ng/mL) Mean ± SEM	*P*-value
NGT group (n = 190)	10.00 ± 0.08	0.0198*
GDM group (n = 95)	8.92 ± 0.26	
T45G SNP		
TT (n = 65 )	9.68 ± 0.21	0.193
TG/GG (n = 220)	9.68 ± 13	
G276T SNP		
GG (n = 161)	9.65 ± 0.15	0.661
GT/TT (n = 124)	9.63 ± 0.15	

*Data show the number of participants in each group, with mean adiponectin level presented in mean ± standard error of mean. NGT, normal glucose tolerance, GDM, gestational diabetes mellitus; Significant if *p-*value is less than 0.05 (*P* < 0.05) using Mann-Whitney *U* test.

### Association of adiponectin levels with clinical characteristics

Since adiponectin hormone has been recognized for its role in various metabolic processes, we also assessed its correlation with various clinical parameters that are involved in the recognition of GDM. Based on our findings, adiponectin levels were negatively correlated with FBS, 1-hour OGTT, 2-hour OGTT, and HOMA-IR (Table 5). Meanwhile, we did not found any interaction between adiponectin levels and anthropometric, lipid, HbA1c, and insulin measurements.

**Table 5:** Relationship between adiponectin and various physical and biochemical characteristics of study participants

Characteristic	Total Adiponectin
Weight	0.062 (0.379)
Height	-0.033 (0.625)
BMI	-0.127 (0.079)
FBS	**-0.250 (< 0.0001)**
1st hour OGTT	**-0.191 (0.001)**
2nd hour OGTT	**-0.225 (< 0.0001)**
Total Cholesterol	0.080 (0.193)
Triglyceride	0.084 (0.171)
HDL	-0.039 (0.523)
LDL	0.071 (0.251)
VLDL	0.085 (0.169)
HbA1c	0.070 (0.254)
Insulin	-0.103 (0.082)
HOMA-IR	**-0.118 (0.046)**

*Data presented are Point-Biserial Correlation Coefficients along with p-values [r (*p-*value)]. Negative correlation coefficient values indicate negative association and positive correlation coefficient values indicate positive association. Correlations are significant if *p-*value is less than 0.05 (*p* < 0.05).

### Association of ADIPOQ gene T45G SNP with lipid parameters, glucose parameters, and measures of insulin resistance

Since GDM is a multifactorial condition, we also evaluated the correlation of ADIPOQ gene SNPs with the clinical characteristics of participants, such as lipid profile, glucose parameters, and insulin resistance. Using point-biserial correlation analysis, we have found out that TG genotype and T allele of T45G polymorphism were correlated with lower levels of total cholesterol, triglyceride, LDL, and VLDL while GG genotype was associated with higher levels of the above mentioned lipid parameters. In terms of glycemic parameters, TT genotype was positively correlated with 1-hour and 2-hour OGTT while G-allele was negatively correlated with these glucose parameters. Contrary, no relationship was found between this polymorphism and other glycemic parameters such as FBS and HbA1c as well as measures of insulin resistance through insulin level and HOMA-IR (Table 6).

**Table 6:** Point-biserial correlation analysis of the ADIPOQ gene T45G SNP genotypes and alleles with physical and biochemical characteristics

Characteristic	Genotype			Allele	
	TT	TG	GG	T	G
FBS	0.068 (0.250)	-0.078 (0.187)	0.023 (0.698)	-0.023 (0.698)	-0.068 (0.250)
OGTT 1st hour	**0.154 (0.009)**	-0.071 (0.229)	-0.064 (0.282)	0.064 (0.282)	**-0.154 (0.009)**
OGTT 2nd hour	**0.122 (0.040)**	-0.047 (0.426)	-0.061 (0.306)	0.061 (0.306)	**-0.122 (0.040)**
Total Cholesterol	-0.042 (0.495)	**-0.185 (0.002)**	**0.241 (< 0.0001)**	**-0.241 (< 0.0001)**	0.042 (0.495)
Triglyceride	0.080 (0.196)	**-0.247 (< 0.0001)**	**0.198 (0.001)**	**-0.198 (0.001)**	-0.080 (0.196)
HDL	-0.055 (0.371)	0.091 (0.138)	-0.049 (0.422)	0.049 (0.422)	0.055 (0.371)
LDL	-0.063 (0.304)	**-0.136 (0.027)**	**0.207 (0.001)**	**-0.207 (0.001)**	0.063 (0.304)
VLDL	0.080 (0.193)	**-0.248 (< 0.0001)**	**0.198 (0.001)**	**-0.198 (0.001)**	-0.080 (0.193)
HbA1c	0.029 (0.634)	-0.069 (0.254)	0.051 (0.406)	-0.051 (0.406)	-0.029 (0.634)
Insulin	-0.096 (0.106)	0.099 (0.097)	-0.020 (0.739)	0.020 (0.739)	0.096 (0.106)
HOMA-IR	-0.092 (0.121)	0.099 (0.094)	-0.024 (0.685)	0.024 (0.685)	0.092 (0.121)

*Data presented are Point-Biserial Correlation Coefficients along with p-values [r (*p-*value)]. Negative correlation coefficient values indicate negative association and positive correlation coefficient values indicate positive association. Correlations are significant if *p-*value is less than 0.05 (*p* < 0.05).

### Association of ADIPOQ gene G276T SNP with lipid parameters, glucose parameters, and measures of insulin resistance

We also observed the association of G276T polymorphism with the above mentioned clinical parameters and based on our results, GT genotype and T allele of G276T polymorphism were negatively correlated with 1-hour OGTT while GG genotype was positively correlated with it. Furthermore, TT genotype and G allele of G276T polymorphism showed a negative and positive correlation, respectively, with HbA1c levels. No correlations however were denoted with the other remaining parameters tested (Table 7). The associations observed between these polymorphisms and GDM parameters in this study were significant but weak.

**Table 7:** Point-biserial correlation analysis of the ADIPOQ gene G276T SNP genotypes and alleles with physical and biochemical characteristics

Parameter	Genotype			Allele	
	GG	GT	TT	G	T
FBS	0.043 (0.473)	-0.021 (0.724)	-0.057 (0.341)	0.057 (0.341)	-0.043 (0.473)
OGTT 1st hour	**0.124 (0.036)**	**-0.119 (0.044)**	-0.016 (0.782)	0.016 (0.782)	**-0.124 (0.036)**
OGTT 2nd hour	0.010 (0.861)	0.009 (0.883)	-0.049 (0.409)	0.049 (0.409)	-0.010 (0.861)
Total Cholesterol	0.003 (0.963)	0.018 (0.768)	-0.054 (0.377)	0.054 (0.377)	-0.003 (0.963)
Triglyceride	0.065 (0.289)	-0.021 (0.728)	-0.115 (0.061)	0.115 (0.061)	-0.065 (0.289)
HDL	-0.011 (0.862)	-0.011 (0.853)	0.057 (0.351)	-0.057 (0.351)	0.011 (0.862)
LDL	0.007 (0.908)	0.011 (0.863)	-0.046 (0.454)	0.046 (0.454)	-0.007 (0.908)
VLDL	0.065 (0.290)	-0.021 (0.727)	-0.115 (0.062)	0.115 (0.062)	-0.065 (0.290)
HbA1c	0.063 (0.304)	-0.006 (0.915)	**-0.141 (0.021)**	**0.141 (0.021)**	-0.063 (0.304)
Insulin	0.056 (0.346)	-0.045 (0.451)	-0.030 (0.612)	0.030 (0.612)	-0.056 (0.346)
HOMA-IR	0.050 (0.401)	-0.038 (0.525)	-0.032 (0.586)	0.032 (0.586)	-0.050 (0.401)

*Data presented are Point-Biserial Correlation Coefficients along with p-values [r (*p-*value)]. Negative correlation coefficient values indicate negative association and positive correlation coefficient values indicate positive association. Correlations are significant if *p-*value is less than 0.05 (*p* < 0.05).

## Discussion

In this present study, the association of two of the most common polymorphisms within the ADIPOQ gene, T45G and G276T SNPs, with GDM was evaluated for the first time amongst selected Filipino pregnant women. Our results showed that none of the ADIPOQ gene polymorphisms showed significant correlation with GDM, adiponectin levels, and measures of insulin resistance. However, there was a significant association between G allele of T45G polymorphism and reduced glucose levels measured on the 1st and 2nd hour OGTT. Similarly, T allele of G276T polymorphism was also negatively correlated with glucose levels on the 1st hour OGTT, while the G allele was positively associated with HbA1c levels.

ADIPOQ gene, which is expressed exclusively in the adipose tissue, specifically encodes for adiponectin hormone that plays a complex role in various hormonal and metabolic processes [40]. Various studies have inversely correlated adiponectin levels with insulin resistance, obesity, metabolic syndrome, and T2DM [19, 33, 41, 42]. Similarly, results of this current study showed that adiponectin levels were negatively correlated with FBS, 1-hour OGTT, 2-hour OGTT, and HOMA-IR, confirming its role on several metabolic processes. Previous studies have also identified that mutations within the ADIPOQ gene affect the level of adiponectin hormone in the blood, compromising its role in maintaining glucose homeostasis [8, 32, 43, 44]. The specific contribution of ADIPOQ-encoded protein to the molecular pathogenesis of GDM however, remains to be expounded as conflicting results have been obtained with regards to their correlations. In this study, although adiponectin levels are significantly decreased in GDM group, the levels are not statistically different according to genotypes of both polymorphisms under study. Several previous studies have reported that hypoadiponectinemia may be predictive of GDM in the early pregnancy [8, 36] and is likewise related to it in mid pregnancy [45] as well as at delivery [46]. Moreover, two separate published studies in Chinese [8] and Malaysian [32] populations have specifically correlated G allele and TG/GG genotype of T45G SNP with low adiponectin levels, demonstrating the role of this SNP on the expression level of ADIPOQ gene. Conversely, in another study conducted on Iranian population, adiponectin level was not associated with GDM and T45G SNP despite the significant correlation found between G allele and TG/GG genotype of T45G SNP and GDM [10]. In our study, adiponectin levels appeared to be a candidate predictive marker for GDM, however, ADIPOQ gene polymorphisms don’t seem to affect its levels in our studied population. These results are similar to a published report in Arab population [13].

Various factors can be considered in evaluating the inconsistent findings regarding the role of these ADIPOQ gene polymorphisms in the development GDM. Most have been linked to disparities in the demographic background, genetic patterns, as well as environmental impacts among various population groups [10]. Another factor that may play a part in the variability of results is the small sample size used in these studies. Meta-analysis on the genetic association of ADIPOQ gene polymorphisms with GDM were previously conducted to administer a more convincing assessment of effect. In one published meta-analysis report conducted in 2016, which included 8 studies, no association was found between T45G SNP and GDM [47]. Meanwhile, in another meta-analysis conducted recently, which included 13 studies, they found significant correlation between T45G SNP and GDM. However, no association was found between G276T SNP and GDM [48]. This conflicting meta-analysis reports indicate the importance of large sample size, in order to produce a more reliable result.

Meanwhile, since T45G SNP in exon 2 is a silent mutation that does not result in an amino acid change (Gly ◊ Gly) and G276T SNP is in intron 2, which is a non-coding region, eliminated by splicing, and is therefore not translated into a final protein product, these SNPs alone may not affect the ADIPOQ gene expression [27], plausibly explaining the negative findings obtained in this present study and other previous studies. Other publications have however postulated that they may be in linkage disequilibrium with other functional polymorphisms [29], thereby affecting various metabolic phenotypes [28, 37, 49]. However, inconsistencies in the results across different population groups may be due to the differences in the LD structures and patterns among several population groups. It is therefore possible that ADIPOQ gene (T45G and G276T) SNPs were not in LD with other functional mutations within this gene leading to the absence of associations with adiponectin levels and GDM in this current study as well as with other metabolic conditions in other previous studies [30, 50]. Moreover, it is also possible that LD structures and patterns in these studied populations don’t have a direct effect on circulating adiponectin levels [30]. Alternatively, according to Komar (2009), silent SNPs do not seem to affect the expression levels of the gene, however they may alter the structural conformation and the biological functions of its protein products [51]. This may explain the lack of association between T45G SNP and adiponectin levels in this study. mRNA expression levels of ADIPOQ gene was however not determined in this study and assumptions were drawn primarily from adiponectin levels in the circulation. It is also possible that adiponectin levels in the serum were not reflective of the levels in the subendothelial space, where they specifically carry out their biological function [52].

There were several limitations in our study. First, our sample size is relatively small, although we used a statistical power of 0.8 and alpha probability error of 0.05 to identify the minimum required sample size. Second, we lack data on environmental factors, such as lifestyle, hindering us from evaluating the effects of interaction between ADIPOQ gene polymorphisms and other factors on GDM. Further study must be conducted to evaluate the gene-environmental interactions in various population groups as this may have an imperative impact on the results. Third, participants in this study were recruited in selected tertiary hospitals and clinics within Metro Manila, and thus may not be representative of the pregnant Filipino women in the Philippines.

In conclusion, ADIPOQ gene (T45G and G276T) SNPs were not directly associated with adiponectin levels and GDM in a Filipino population, although weak associations were found between these SNPs and some of the GDM-related blood analytes. Nonetheless, we recommend that more samples should be included in future studies. Furthermore, screening of additional polymorphisms within the ADIPOQ gene is also deemed necessary to further explain the combined functional effects of various polymorphisms within this gene on GDM risk as well as on various metabolic phenotypes that share or contribute to the pathogenesis of this condition.

## Data Availability

The datasets used and/or analyzed during the current study are available from the corresponding author on reasonable request. Data can be accessed at https://bigd.big.ac.cn/gvm/getProjectDetail?Project=GVM000553.
